# Physical capacity is associated with attention but not with general executive function in middle aged adults

**DOI:** 10.1371/journal.pone.0321450

**Published:** 2025-05-05

**Authors:** Khalil Iktilat, Michal Isaacson, Roy Tzemah-Shahar, Maayan Agmon

**Affiliations:** 1 Department of Gerontology, Faculty of Health and Social Welfare, University of Haifa, Haifa, Israel; 2 Ramat Gan Academic College, Israel; 3 Faculty of Health and Social Welfare, The Cheryl Spencer Institute for Nursing Research, University of Haifa, Haifa, Israel; University of North Texas Health Science Center, UNITED STATES OF AMERICA

## Abstract

Associations between Physical Capacity (PC) and various aspects of executive function, including attention, have been identified in older and younger cohorts. Yet, the relationship among middle-aged individuals at the gateway of aging is largely understudied. The primary objective of this study was to investigate the relationship between physical capacity indicators and attention, a key component of executive functions, in middle-aged Muslims in Israel, a minority group experiencing chronic health disparities. This cross-sectional study included 255 participants (159 women, 96 men), aged 51.29 ± 4.26 (45–60 years). PC was assessed using the six-minute walk test (6MWT) and the 30-second sit-to-stand test (30 STS). Attention and executive function were assessed with the Stroop C Test and Trail Making Test (TMT) respectively. We found a statistical trend suggesting a positive correlation between the 30 STS and Stroop C scores, when controlling for age, sex, education level, and hemoglobin A1c, indicating that higher physical capacity is associated with better selective attention and response inhibition (β = 0.132, P = 0.057). In contrast, no correlation was found between the 30 STS and the TMT-B (β = −0.107, P = 0.107), suggesting that alternating attention and overall executive function are not associated with physical capacity in this cohort. Interestingly, neither functional assessment was associated with the 6MWT, suggesting that among healthy, free living, middle-aged individuals, a more challenging assessment, like the 30 STS may be most relevant when examining associations between physical capacity and executive function. Our findings highlight the nuances of the relationship between physical capacity and aspects of executive function in this demographic. These results add to the body of literature associating physical capacity to selective attention but not executive function in middle-aged, minority populations. Further extension of these findings can support healthy aging, ultimately enhancing quality of life and reducing morbidity in older age.

## Introduction

Executive functions are higher-order cognitive abilities that enable planning, initiating, monitoring, and completing complex tasks [1]. These functions include self-control, cognitive flexibility, working memory, attention, and planning [2] which are crucial for daily functioning, academic and occupational success, and overall quality of life [3,4]. Executive functions, particularly attention, begin to decline in early middle age, and in some cases as early as 20–40 years of age [5–8], with long-term impacts on various aspects of life, including increased risk of falls and reduced ability to perform complex tasks in older age [9].

Attention resources, a component of executive function, are essential for maintaining productivity, managing the increasing complexity of life’s tasks, and preventing falls [9]. Declines in attention can lead to difficulties in multitasking, distractedness, and overall reduced cognitive efficiency, significantly impacting both personal and professional life [10]. Attention can be further categorized into several types: selective attention, which involves focusing on relevant stimuli while ignoring distractions; sustained attention, the ability to maintain focus over prolonged periods; divided attention, the capacity to manage multiple tasks simultaneously; alternating attention, which allows for rapid shifting between tasks; and executive attention, which involves monitoring and resolving conflicts among thoughts and actions [11–13]. These types of attention are interrelated and often overlap in real-world tasks. For example, walking in complex environments requires various types of attention, like selective attention to focus on the path while ignoring irrelevant stimuli, such as distant conversations or passing vehicles, and divided attention to manage multiple tasks like adjusting pace and direction while responding to traffic signals and avoiding obstacles [14].

Environmental and individual factors have been associated with attention and overall executive function. Particularly, among middle aged individuals, subjective socioeconomic standing is positively associated with executive function [15] and brain functional network connectivity and morphology [16], which could underlie the previously reported differences in executive function across socioeconomic standings. Psychological distress, prevalent in middle-aged adults due to various life demands, has also been associated with impairments in executive functions, including attention, potentially affecting cognitive performance and daily functioning [17,18]. Furthermore, external factors like poverty and community violence can influence both psychological distress and executive functions [19]. Increasingly, research has also tied physical capacity to attention, and mounting research suggests that increasing various components of physical capacity, and particularly dual-task abilities, can increase attention as well as processing speed [20]. This relationship is bidirectional – for example, improved attention can also improve gait performance and reduce fall risks [21].

The underlying mechanisms that positively link physical capacity with executive function and attention can be related to improved blood flow to the brain, reduced inflammation, and promotion of neuroplasticity that are achieved with increased physical capacity [22], often measured indirectly through C-reactive protein (CRP) [23,24] and *hemoglobin A1c* (HbA1c) profiling [25,26]. Both CRP and HbA1c are widely recognized as cardiometabolic biomarkers associated with some of the most prevalent non-communicable diseases, including cardiovascular disease and type 2 diabetes [27]. Most research on this association is conducted among older individuals [28–31] or across wide age ranges (e.g., [32]). Focusing specifically on middle-aged individuals can fill an important research gap, uncovering nuances unique to this age-group [33]. For example, in a meta-analysis of tens of studies focusing largely on older adults over 60, higher levels of physical capacity were linked to better performance in selective and sustained attention [20]. Similarly, a study on community-dwelling older adults with a mean age of 72.7 (SD 5.9) revealed that decreases in attention and general executive function were associated with lower physical capacity. Interestingly, different physical capacity tasks exhibited distinct relationships with executive function; for example, walking while talking was not associated with improvements in higher-level cognitive function, while both walking with obstacles and a fast walk were associated with improvements in cognitive function [34]. In another study of post-stroke older adults (mean age: 69.1 ± 9.4), there was a positive correlation between complex gait tasks and executive function, including tasks related to attention, but this correlation was reduced when considering basic gait tasks [35]. Because middle-aged individuals are largely mobile, independent, and relatively healthy, measures of capacity used among older (potentially frail or sedentary) individuals might not be appropriate to tease out fine-scale differences, and this could explain why patterns of physical capacity-attention relationships are not conserved across studies in this age group.

Because executive function and physical capacity tend to deteriorate during the aging process, understanding the relationships between them in this age group is of high relevance. Accordingly, the purpose of the present study is to examine this relationship in middle-aged adults (45–60 years) in a unique environment with heightened exposure to violence. By situating our research within this distinct population, we aim to contextualize how environmental stressors and demographic factors may shape the trajectory of cognitive and physical aging. Examining deviations from established normative data can further elucidate whether these influences accelerate or modify typical age-related changes, providing critical insights for future interventions.

## Materials and methods

### Cohort recruitment

**Fig 1:**
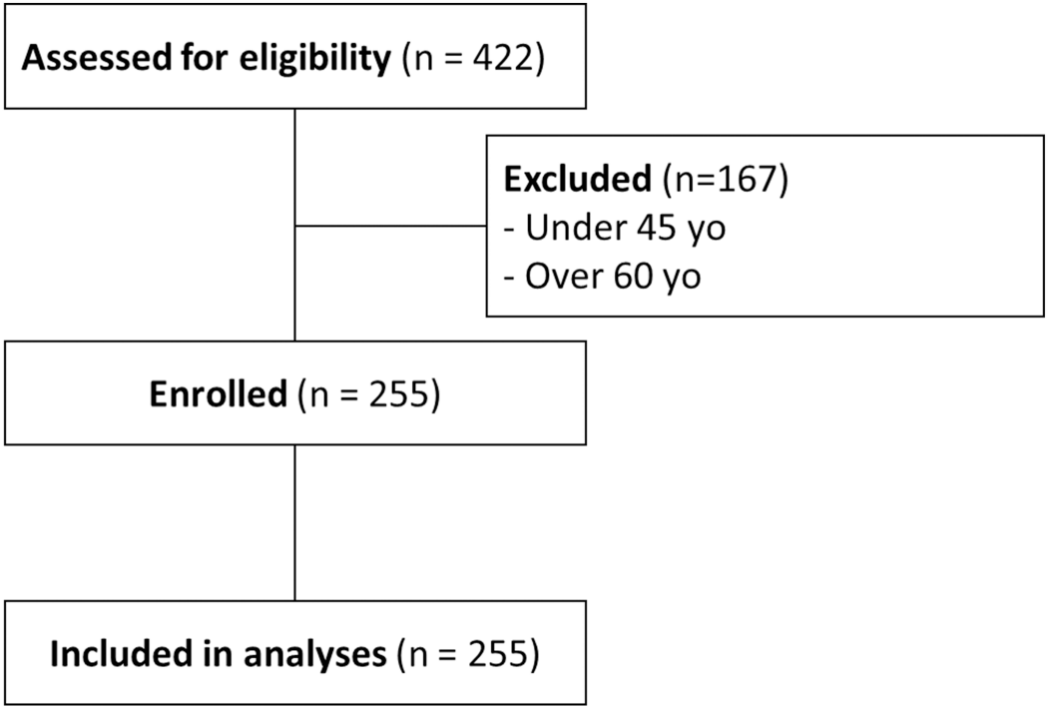
Flow chart of participant recruitment and enrollment.

This research is part of a larger cross-sectional study of middle-aged adults. Recruitment took place in three Muslim villages in northern Israel from 01/03/2021–30/09/2021, utilizing a website/mobile app, public posters, and word-of-mouth referrals. The inclusion criterion was age 45–60 years old; exclusion criteria included any history of neurological conditions (such as strokes or other neurological disorders) or major visual/hearing impairments, as well as acute medical issues (such as joint pain, back pain, or other acute illnesses) and mobility and cognitive disabilities (Fig 1). The study received approval from the Ethics Committee of the Faculty of Social Welfare and Health Science at the University of Haifa, Israel (approval number: 237/21).

### Cohort characteristics

Data collection occurred over two sessions: the first session lasted one hour, during which participants received study information, signed consent forms, and provided various physiological measures. They also filled in surveys and performed physical capacity and executive functions tasks (described below). The second session, one week after the first, was 15 minutes during which fasting blood samples were taken.

### Executive function assessments

The Color-Word Stroop Test [36] measures selective attention and response inhibition. In the test, the participant is asked to state the color of each word. There are three parts to this test, but only scores from part C (here referred to as Stroop C) were included in models because it is the most cognitively challenging (higher level of selective attention and response inhibition) [37]. The three parts are (A) a sheet of color words, with each word printed in black ink, (B) a sheet where each word is printed in a matching ink color, and (C) the interference part in which color words are printed in an ink color that does not match the word (i.e., the word red is printed in blue). We counted the number of correctly named words in a 45 second testing period. Correctly named colors and executive function are positively correlated. Previous research on middle-aged individuals found Stroop C scores ranging between 40 and 50 words [36,38].

The Trail Making Test (TMT, here the TMT-B) [39] was used to measure executive function. Specifically, it measures alternating attention, complex visual scanning, motor speed, and cognitive flexibility. Participants draw a line connecting alternating, randomly positioned numbers and letters as quickly as possible without lifting their pencil from the paper [40], and the time to connect a full set (page) of numbers and letters is recorded. Here, we only used part B (TMT-B) [39,41], in which the participant is required to alternately connect circled numbers from 1 to 13 and circled letters from A to N (e.g., 1-A-2-B-3-C and so on). Our choice was based on well-established literature demonstrating that TMT-B provides a more comprehensive assessment of executive function, particularly cognitive flexibility, set-shifting, and divided attention, while TMT-A primarily evaluates basic processing speed and visual search ability [41–43]. Shorter time-to-completion is indicative of higher executive function. In middle aged individuals, times to completion typically ranged from 45-80s [39,44].

### Physical capacity indicators

The 6-minute walk test (6MWT) is a common test to assess fitness abilities, suitable for a wide range of populations and requiring minimal equipment [45,46]. Participants were asked to walk “as fast as they can without running”, at a speed that would allow them to continue walking for 6 minutes, back and forth between two ends of a 10-meter walking trail. The test was conducted indoors, on a flat floor, in halls of the community centers where participants were recruited. Total distance walked was measured for each minute separately and for the six minutes cumulatively and divided by time walked, respectively, to receive average walking speeds. The walking trail was marked on the floor using colored tape, with additional markers every 1 meter to allow for exact measurements. In our analyses, measurements from the total six minutes were used as a measure of endurance that represents walking speed under aerobic stress [47,48]. In similarly aged cohorts, individuals typically walked 344-376m [46,49].

The 30 second sit-to-stand test (30 STS) assesses physical fitness and can predict physical capacity, serving as a measure of functional performance [46]. During the test, the subject is instructed to sit on a standard chair (45 cm height, straight back) and then stand, repeating this action as many times as possible for 30 seconds. The subject crosses their hands on their chest, and the number of the subject’s repetitions is recorded. The test was examined and validated in a previous study with a similar population [46]. Typically, middle-aged participants completed 10–17 repetitions [50–53].

### Covariates: demographic, metabolic, inflammatory markers, and psychological distress

Age, sex, height and weight were collected, allowing for body mass index (BMI) calculation, and waist and hip circumferences were measured. Other demographic data (e.g., education level, socioeconomic status) and health behaviors (e.g., smoking status) were also collected using self-administered questionnaires. Socioeconomic status was rated using a single question focusing on subjective income level in comparison to the national average, using a five-point Likert scale ranging from 1 (“significantly lower than the average”) to 5 (“significantly higher than the average”). Education level was measured as having completed at least an associate degree (0/1: no/yes). Psychological distress was assessed using the Kessler 6 [54] survey as was recently described for this cohort [55]. These variables were considered because they have previously been found to have associations with executive function [56]. Then, blood pressure was measured using a standard electronic blood pressure device (Seca, Hamburg, Germany), before physical capacity tests, and after subjects rested for five minutes, with their legs uncrossed and touching the floor. Three consecutive measurements were taken, and average systolic and diastolic pressure were calculated. Additionally, glucose and triglyceride levels from fasting blood tests were recorded. We used HbA1c levels as a metabolic marker [25,26]; additionally, CRP concentration was used to assess inflammation [23,24].

For all blood tests, blood was collected after an 8–12 hour fast from an antecubital vein into a 3.8% sodium citrate-containing tube. Blood was stored at -80°C until assayed. Blood diagnostics were performed in Holy Family Hospital, Nazareth, Israel in an established reference laboratory under quality control supervision. Blind duplicate samples (5%) were used to estimate the analytic variation within runs and over time [57]. CRP was measured using an Abbott Architect CI-4100 (Abbott, Germany), reagent no. 8-6K26-30, with a detection range of >0.6 mg/dl.

### Statistical analysis

Formal analysis of results was conducted using IBM SPSS v.27.0.0.0; the level of significance was set at P < 0.05 and P < 0.10 was referred to as a statistical trend. Variables are presented as mean ± SD (standard deviation) and outliers (beyond two standard deviations of the mean) were excluded. Bivariate correlations were examined to determine which demographic, metabolic, and inflammatory factors were correlated with 30 STS, 6MWT, and executive function (Pearson correlation coefficient) and then controlled for in linear regression models.

To address our hypothesis that physical capacity indicators are associated positively with Stroop C and negatively with TMT-B (executive function indicators) among middle-aged adults, linear regression models, adjusted for age, sex, education, HbA1c, CRP, and self-reported psychological distress (Kessler 6) (based on findings from bivariate correlation analyses), were built for each fitness parameter and executive function indicator separately (Stroop C – 30 STS, Stroop C – 6MWT, TMT-B – 30 STS, and TMT-B – 6MWT).

## Results

A total of 255 participants (159 women, 96 men), aged 51.29±4.26 (45–60 years) were included in the final analysis. In some cases (indicated in tables) data were incomplete; for each model, only those participants with complete data were included. Demographics and distributions of the main variables are presented in Table 1.

**Table 1:** Descriptive statistics of the middle-aged Muslim cohort.

	Total cohort	Men	Women	P value
**Sample size (%)**	255	96 (37.6%)	159 (62.4%)	–
**Age**	51.29 ± 4.26 (45–60)	51.58 ± 4.33 (45–60)	51.11 ± 4.22 (45–60)	0.395
**Income** ^2^	2.57 ± 1.1 (1–5)	3.12 ± 1.04 (1–5)	2.19 ± 0.97 (1–4)	**<0.001** ^3^
**Stroop C**	37.95 ± 11.56 (5–77)	38.04 ± 10.15 (16–70)	37.89 ± 12.36 (5–77)	0.921
**TMT-B**	89.43 ± 33.43 (25–212)	88.47 ± 34.11 (37–212)	89.43 ± 33.43 (25–212)	0.720
**30 STS**	15.23 ± 4.04 (6–30)	16.51 ± 4.33 (7–30)	14.46 ± 3.66 (6–27)	**<0.001** ^1^
**6MWT**	537.26 ± 111.30 (106–968)	580.417 ± 98.11 (350–924)	511.04 ± 110.94 (106–968)	**<0.001** ^1^
**Education (category)**	(N = 227)	(N = 86)	(N = 141)	**0.027** ^1^
** Academic**	42.4%	51%	37.1%	
** Non-academic**	46.7.9%	38.5%	51.6%	
**Kessler 62**	1.89 ± 0.74	1.77 ± 0.68	1.96 ± 0.77	**0.0463**
**CRP**	4.69 ± 0.66 (0.60–33.80)	3.42 ± 2.56 (0.60–16.02)	5.46 ± 5.62 (0.60–33.80)	**<0.001** ^1^
**HbA1c**	5.69 ± 0.66 (4.86–10.33)	5.65 ± 0.51 (4.88–8.11)	5.72 ± 0.74 (4.86–10.33)	0.479^1^
**BMI**	30.51 ± 5.02 (19.54–56.54)	30.27 ± 4.15 (22.09–43.12)	30.66 ± 5.49 (19.54–56.51)	0.548^1^
**Waist circumference**	95.75 ± 12.55 (61–137.50)	100.05 ± 11.24 (74–127)	93.15 ± 12.62 (61–137.50)	**<0.001** ^3^
**Hip circumference**	106.37 ± 10.14 (78.50–147)	105.15 ± 9.05 (84.5–120.5)	107.11 ± 10.70 (87.50–147)	0.137
**Systolic BP**	124.06 ± 17.35 (78–208.67)	131.86 ± 16.55 (101.33–208.67)	119.36 ± 16.13 (78–172.5)	**<0.001** ^1^
**Diastolic BP**	83.21 ± 9.82 (55–118.33)	84.43 ± 9.55 (55–118.33)	82.47 ± 9.93 (57.67–114.67)	0.123
**Active Smokers**	19.6%	40.6%	6.9%	**<0.001** ^4^

Characteristics of the cohorts were compared between men and women with relevant statistical tests. Statistically significant values are bolded. Data are presented as mean±SD (range), 30 STS: 30 seconds sit-to-stand test, 6MWT: six-minute walk test.

^1^T-test.

^2^Likert scale: 1–5, 5 highest.

^3^Mann-Whitney U Test.

^4^Fisher exact test.

**Fig 2:**
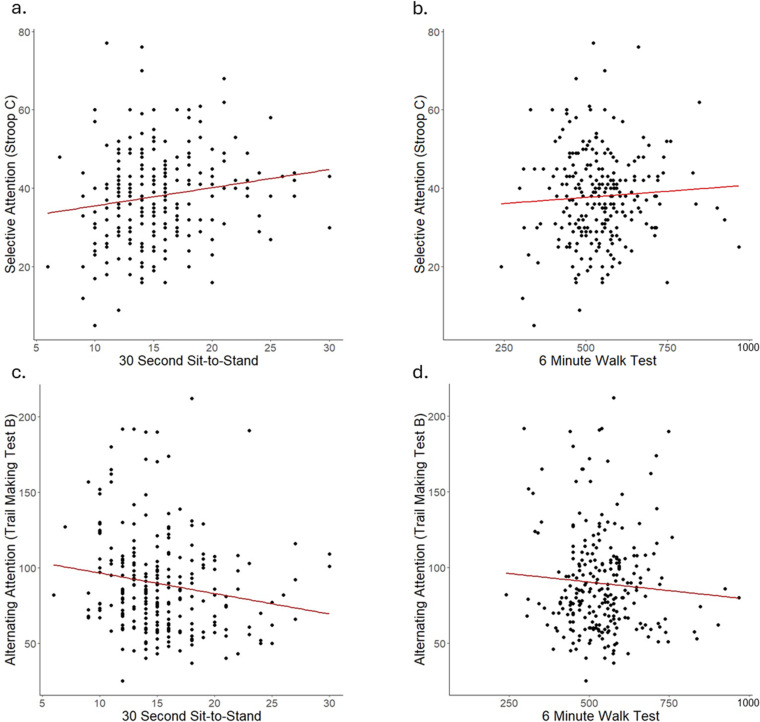
Correlations between physical capacity metrics and measures of attention. Pearson’s correlation between selective attention, measured by the Stroop C assessment and (a) the 30 second sit-to-stand test (r = 0.163, P = 0.010) revealed a significant correlation, but its correlation with (b) the six minute walk test (r = 0.058, P = 0.358) was not significant. Pearson’s correlation between alternating attention, measured by the trail marking test B and (c) the 30 second sit-to-stand test (r = −0.164, P = 0.009) revealed a significant correlation, but no correlation was observed with (d) the six minute walk test (r = −0.073, P = 0.248). When examining correlations after including covariates (Table 3), only the relationship between selective attention and the 30 second sit-to-stand showed a trend towards significance (β = 0.132, P = 0.057).

Not surprisingly, the two measures of executive function and the two measures of fitness were significantly correlated (Stroop C – TMT-B: r = −0.233, P-value < 0.001; 30 STS – 6MWT: r = −.337, P-value < 001). When examining correlations between fitness and executive function measures, the 30 STS, but not the 6MWT, was significantly correlated with both executive function measures (positively with Stroop C (r = 0.163, P = 0.010), and negatively with TMT-B (r = −0.164, P = 0.009); Table 2 and Fig 2).

**Table 2:** Bivariate correlations of covariates with executive function (Stroop C and TMT-B) and physical capacity indicators (30 STS and 6MWT).

	Stroop C	TMT-B	30 STS	6MWT
**Stroop C**	**--------**	-0.233***	0.163**	0.058
**TMT-B**	−0.233***	--------	−0.164**	**−**0.073
**30 STS**	0.163**	−0.164**	--------	0.337***
**6MWT**	0.058	**−**0.073	0.337***	--------
**Education**	0.239***	−0.295***	0.132*	0.101
**Age**	**−**0.068	0.197**	−0.172**	**−**0.048
**CRP**	−0.124*	0.058	**−**0.144	−0.203***
**HbA1c**	**−0.162**	0.19**	−0.128**	−0.132*
**Sex**	**−**0.006	0.023	−0.245***	−0.303***
**BMI**	**−**0.092	0.077	−0.179**	−0.181**
**Kessler 6**	**−**0.040	0.145*	**−**0.122	−0.204***

Significant Pearson correlations are in bold. SES: socioeconomic status, Stroop C and TMT-B: measures of executive function, 30 second sit to stand and six-minute-walk: measures of physical capacity, HbA1c and CRP: metabolic and inflammatory markers, Kessler 6: self-reported measure of psychological distress.

* p < 0.05.

** p ≤ 0.01.

*** p ≤ 0.001.

**Table 3:** **Linear regression modes**.

Dependent Variable	Independent Variables	Standardized β	P-value
**Stroop C (n = 221)**	1. Sex	0.045	0.508
	2. Age	0.016	0.812
	3. Education	0.213	**0.002**
	4. HbA1c	−0.069	0.314
	5. 30 STS	0.132	0.057
**Stroop C (n = 221)**	1. Sex	0.039	0.579
	2. Age	0.003	0.962
	3. Education	0.220	**0.002**
	4. HbA1c	−0.067	0.344
	5. CRP	−0.082	0.245
	6. Kessler 6	0.004	0.949
	7. 6MWT	0.021	0.765
**TMT-B (n = 222)**	1. Sex	−0.047	0.471
	2. Age	0.127	0.054
	3. Education	−0.250	**<0.001**
	4. HGA1C	0.150	**0.023**
	5. 30 STS	−0.107	0.107
**TMT-B (n = 222)**	1. Sex	−0.036	0.593
	2. Age	0.155	**0.019**
	3. Education	−0.246	**<0.001**
	4. HbA1c	0.138	**0.042**
	5. CRP	−0.005	0.939
	6. Kessler 6	0.116	0.076
	7. 6MWT	−0.032	0.636

Significant findings are in bold and trends towards significance are underlined. This model is adjusted for sex, age, education, and HbA1c, as well as CRP and psychological distress (with the models that include 6MWT).

We next examined associations between executive function and demographic, metabolic, and inflammatory markers towards identifying important covariates to include in our models. We found that education was significantly negatively associated with TMT-B and positively associated with Stroop C. Furthermore, TMT-B was significantly positively correlated with age and HbA1c levels and Stroop C was significantly negatively correlated with CRP and HbA1c levels. We also found a significant positive correlation between self-reported psychological distress and TMT-B but no correlation was observed with the Stroop C.

Lastly, we examined correlations between physical capacity parameters and covariates to understand the relationships between these predictors. HbA1c (r = −0.128, P = 0.043), BMI (r = −0.179, P = 0.004), age (r = −0.172, P = 0.006), and sex (r = −0.245, P =< 0.001) were significantly negatively correlated with 30 STS, while education (r = 0.132, P = 0.047) was positively related. It is worth noting that no significant relationship was found with between 30 STS and CRP or Kessler 6, therefore they were not included in the regression model test that includes the 30 STS. Like with the 30 STS, the 6MWT was significantly and negatively correlated with HbA1c (r = −0.132, P = 0.036), BMI (r = −0.181, P = 0.004) and sex (r = −0.303, P =< 0.001), as well as CRP (r = −0.203, P = 0.001) and self-reported psychological distress (Kessler 6; r = −0.204, P = 0.001). There were no significant correlations between 6MWT and age or education.

Following bivariate correlation analyses, linear regression models to determine the relationship between 30 STS or 6MWT (Table 3) and executive function were built controlling for sex, age, education, and HbA1c (as well as CRP and Kessler 6 in the models that include 6MWT). The 30 STS was correlated (statistical trend) with Stroop C (β = 0.132, P = 0.057), as was education (β = 0.213, P = 0.002). Compared with the 30 STS, we did not find effects of the 6MWT on Stroop C (β = 0.018, P = 0.793). When examining the association between these physical capacity parameters and TMT-B, no significant correlations were identified. For TMT-B, other significant variables included education (β = −0.250, P < 0.001; β = −0.285, P < 0.001) and HbA1c (β = 0.150, P = 0.023; β = 0.141, P = 0.037), indicating that both lower education and higher HbA1c were associated with worse performance on the TMT-B. Additionally, age was a significant predictor in the model that included 6MWT (β = 0.143, P = 0.031).

## Discussion

The current study aimed to explore the relationship between physical capacity and aspects of executive functions, specifically attention, in a middle-aged cohort. This age group is at a pivotal life stage where both physical and cognitive declines can be identified and treated [58]; however, this age group is largely understudied. Among our study cohort, measures of physical capacity were similar to or greater than similarly aged cohorts previously characterized [45,46,49,51,52]. The average number of STS in our cohort was 15.23, at the upper end of the range for similarly aged individuals, and the average meters walked was 537.26, beyond that typically recorded for individuals aged 45–60. This could be a result of strict exclusion criteria during recruitment; further research is needed to understand if this is a representative characteristic of the study population or a consequence of the sampling method. Surprisingly, despite high physical capacity in this cohort, attention measures were lower than other cohorts of middle-aged individuals [36,38,39,44]. One factor underlying this pattern could be associated with the study population’s heighted exposure to violence [59], a pressure known to affect various components of attention [60,61]. Similarly, self-reported psychological distress, stemming from exposure to violence or other environmental stressors, may lead to attention deficits in this community (TMT-B times were significantly increased among those that reported higher distress (r = 0.145, P = 0.023). Previous work has also suggested that psychological distress associated with a sense of insecurity, including fear of leaving the house, can impact both physical capacity and attention [62,63].

Our findings suggest there may be a positive correlation between physical capacity, measured by the 30 STS, and both alternating and selective attention assessed by Stroop C and TMT-B, respectively. When controlling for covariates, though, only the association between the challenging physical capacity task and selective attention was preserved, reflecting better response inhibition among those with higher physical capacity. Alternating attention and general executive function were not associated with either physical capacity measure, when considering covariates (age, sex, education, HbA1c, CRP, and distress). The non-significant association between CRP and measures of executive function in our full model could be due to the nature of the markers themselves and due to the profile of our cohort. The specificity of these markers has been debated, particularly in relation to their ability to predict cognitive outcomes independently of other health factors [64,65]. Furthermore, our cohort is composed of largely healthy individuals, a population in which we do not expect large variations in CRP and HbA1c, as these markers tend to be more predictive of cognitive dysfunction in older adults or those with metabolic disease [66].

Our findings are partially in line with [29], who reported that executive attention in older adults [70–98] could significantly predict gait characteristics (e.g., velocity and cadence). Similarly, a study of older women aged 70–80 showed significant correlations between basic daily life activities (e.g., walking, climbing steps, putting on pants) and executive attention, as well as with spatial learning, and spatial memory [67]. Unlike in our study, other components of executive function, namely memory and verbal IQ, in the former study [29], and spatial processing in the latter [67], were also correlated with physical capacity among older individuals. We did not find a connection between physical capacity and alternating attention and overall executive function highlighting that patterns observed across several studies in older individuals do not characterize middle-aged individuals.

An alternative explanation could be that differences in the relationships between aspects of attention or executive function and physical capacity do not necessarily vary by age-group (our middle-aged cohort vs previously studied older cohorts) but rather by population, based on other, yet unidentified, factors. While we did not find a significant effect of distress in our models, other population-specific factors may be at play. Several studies in older populations also presented results which were very similar to ours, supporting the continuity of capacity-attention relationships from middle-age and into later stages of life. In a study conducted in Brazil on 76 institutionalized older adults aged 65 and above, researchers found that poor physical capacity was associated with impaired attention but was not significantly related with other executive functions [68]. Similarly, research in South Korea on 94 healthy older women aged 62–86 demonstrated a significant association between cardiovascular endurance and attention, but no significant relationship with other cognitive functions [69]. The differences between findings from [29] and [67] – which found correlations between capacity and multiple aspects of executive function – and this body of evidence, which is in line with our findings that only selective attention is correlated with physical capacity, highlight the nuances of the interplay between executive function components and physical capacity when studying adult populations. These inconsistent findings in overlapping age groups support the need for standardized research in narrow target populations, like the research we present here, to ensure reproducibility and generalizability. Taking this even further, research by [70] on a cohort of younger adults, with a narrow age range [38–43], did not reveal any associations between physical activity (subjective or objective), and cognitive functions, including executive functions. Continued, focused research is important for elucidating age-specific, cross-cohort patterns.

Beyond examining the connection between attention and executive function with physical capacity, when examining our full statistical models, including covariates, we saw that not all components of executive function are significantly correlated with age. While selective attention (Stroop C test) was not correlated with age, considering a more complex mental flexibility requirement, represented by the TMT-B test, revealed age-associated effects, suggesting that executive function and alternating attention decreased with age in our cohort. This could signify that in middle-age, before physical capacity truly begins to decline, age alone might be a better predictor of executive function.

Our analyses including covariates (Table 3) revealed that education level was significantly positively associated with both types of attention among our middle-aged participants. Higher educational attainment, as highlighted in the literature, is consistently linked to enhanced executive functions, particularly selective and alternating attention [44,71,72]. Beyond education, additional covariates such as income, cardiovascular health, smoking, systolic blood pressure (BP), and body mass index (BMI) also warrant attention due to their distinct contributions to physical capacity and executive function. Income disparities, for example, may influence cognitive and physical health through differential access to health-promoting resources and chronic stress exposure [16]. Poor cardiovascular health and smoking—more prevalent among men in our cohort—are associated with systemic inflammation and reduced cerebral perfusion, which negatively impact both cognitive and physical performance [32,73]. Moreover, elevated BMI contributes to metabolic dysregulation and chronic inflammation, further linking it to poorer executive function and reduced physical performance [25,26]. While these variables were statistically controlled in our models, their complex interplay highlights the need for more nuanced analyses, particularly stratified by sex, to explore their moderating effects on the relationship between physical capacity and executive function in middle-aged adults.

Here, we also show how some measures of physical capacity are more informative than others among middle aged individuals. We found a connection between 30 STS and attention but not 6MWT and attention, likely due to the increased physical demand of the former. The 30 STS is characterized by short duration and focused muscle engagement, requiring participants to use both speed and strength to rapidly transition from sitting to standing repeatedly, whereas the 6MWT involves walking, which is a relatively low intensity activity, and may not sufficiently stimulate the acute physiological responses necessary to impact attention-related executive functions [46]. Accordingly, higher intensity, mixed task activities, like the 30 STS test, may require greater attention explaining the positive relationship between selective attention and this task that we observed in our cohort. In support of this, the 30 STS measures subtle changes in lower extremity strength and balance—factors closely linked to cognitive function—rendering it a more sensitive instrument for assessing attention-related cognitive decline in various populations [74–76]. These findings support assessment of complex capacity tasks, at least in studies of middle-aged adults for whom basic physical capacity has not yet begun to decline (age and 6MWT were not correlated). We recommend conducting further studies across the lifespan, and on diverse populations ‘with unique environmental pressures (socioeconomic status, health status, field of employment, education levels), to validate these findings, understand if the middle age period is truly unique, and determine if a single physical capacity test can be used longitudinally throughout life or if age-tailored assessments are necessary.

One primary limitation of this study is its cross-sectional design, which restricts the ability to infer a causal effect of physical capacity on attention. Additionally, individuals with acute pain, such as back or knee pain, which are common in this age group, were excluded from the sample, potentially limiting the generalizability of our findings to the broader middle-aged population. Furthermore, the reliance on specific tests for physical capacity (6MWT and 30 STS) and attention (Stroop Test and Trail Making Test) might not capture all relevant aspects of physical capacity and executive functions. As demonstrated here, our cohort is also characterized by higher physical capacity and lower attention than previously reported for this age-group, highlighting that our results should be further explored for generalizability. Furthermore, related biomarkers and underlying mechanisms connecting physical capacity and attention were not examined here. Addressing these limitations in a multi-cohort follow-up experiment could validate that the trend we observed here is representative of middle-aged individuals in broader contexts.

Another limitation when interpreting findings from this study is the demographic sex differences in our cohort (Table 1). Specifically, income, cardiovascular health indicators, smoking status, systolic blood pressure, body mass index, and psychological distress levels differed between the sexes. While sex and other covariates were statistically controlled, their importance in the relationship between physical capacity and executive function merits further exploration. Socioeconomic factors, such as income, have been shown to influence cognitive function and physical health [16]. Similarly, poor cardiovascular health and smoking, which were more prevalent in men in our cohort, are known to affect both physical capacity and cognitive function [32,73,77]. These factors may contribute to the observed sex differences in physical capacity and potentially moderate, or even mask, the relationship between physical capacity and executive function. Thus, when sample sizes allow, we suggest stratifying analyses by sex to elucidate the complex interplay between these variables and sex, physical capacity, and executive function in middle-aged adults.

Future research should employ longitudinal designs on larger cohorts and expand the demographic scope to include diverse populations. Incorporating a broader range of physical capacity and executive function assessments would provide a more comprehensive understanding of the interactions between physical and cognitive health in this transitional period in life and lend further support to our findings. Additionally, investigating underlying mechanisms such as neuroplasticity, brain blood flow, and inflammation could reveal how physical capacity influences executive functions. Intervention studies aimed at improving physical capacity in middle-aged populations are also recommended to assess their potential in mitigating cognitive decline and maintaining cognitive health during aging. Future studies should also aim to collect comprehensive participant medical background data, including detailed information on diabetes status, inflammatory and metabolic medication use, and disease duration. This will enhance the interpretation of future findings and allow for better adjustment of potential confounders in the analysis of cognitive function and physical capacity. Additionally, future research should consider using a panel of specific inflammatory and metabolic markers, rather than relying on a single inflammatory index, to better capture the complex biological pathways linking systemic inflammation to cognitive function.

In conclusion, our study highlights the positive relationship between physical capacity and attention but not overall executive function in middle-aged adults. These associations should be further examined in similar cohorts, studied in this specific window of the aging process, to understand this connection mechanistically and develop effective widespread interventions that meet the goal of preventing premature cognitive decline and promoting healthier aging. Furthermore, exploration in cohorts with other unique environmental pressures can help to further elucidate the generalizability of patterns across populations.
